# CXCL14 as an emerging immune and inflammatory modulator

**DOI:** 10.1186/s12950-015-0109-9

**Published:** 2016-01-05

**Authors:** Jing Lu, Mita Chatterjee, Hannes Schmid, Sandra Beck, Meinrad Gawaz

**Affiliations:** Medizinische Klinik III, Kardiologie und Kreislauferkrankungen, Universität Tübingen, Otfried-Müller-Strasse 10, 72076 Tübingen, Germany

**Keywords:** CXCL14, Migration, Antimicrobial activity, Infection, Inflammation

## Abstract

CXCL14, a relatively novel chemokine, is a non-ELR (glutamic acid-leucine-arginine) chemokine with a broad spectrum of biological activities. CXCL14 mainly contributes to the regulation of immune cell migration, also executes antimicrobial immunity. The identity of the receptor for CXCL14 still remains obscure and therefore the intracellular signaling pathway is not entirely delineated. The present review summarizes the contribution of CXCL14 in these two aspects and discusses the biological mechanisms regulating CXCL14 expression and potential CXCL14 mediated functional implications in a variety of cells.

## Background

**Fig. 1:**
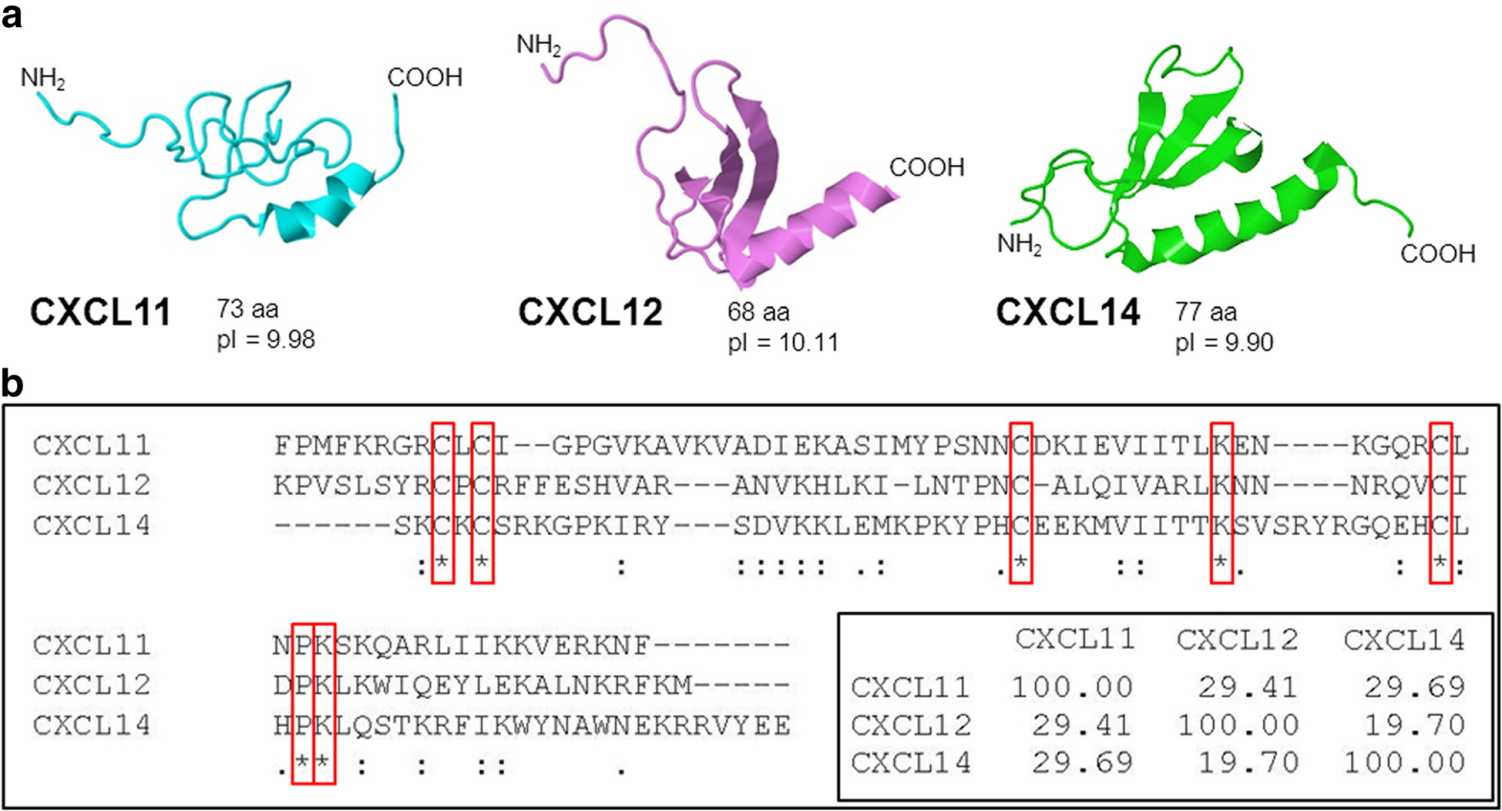
Structural similarities of CXCL11, CXCL12 and CXCL14. **a** CXCL11, CXCL12 and CXCL14 are all small chemokines with a similar molecular structure. The three chemokines all share a C-terminal α-helix and comprise high pI values. Structures of the molecules were obtained by RCSB protein data bank and pdb-files were displayed with Jmol: an open-source Java viewer for chemical structures in 3D. http://www.jmol.org/. **b** CXCL11, CXCL12 and CXCL14 share high amino acid sequence conservation with 4 cysteines, 2 lysines and 1 proline residue being conserved throughout all 3 chemokines (conserved amino acids shown by asterisk *). Alignment was performed using Clustal Omega multiple sequence alignment tool. (References: [12, 65–67])

Chemokines are 8–14 kDa chemoattractant cytokines mainly regulating cell migration that play an important role in immune surveillance, inflammation, and cancer [1–3]. Chemokines induce intracellular signaling through G protein-coupled cell-surface receptors [3]. They contain several (usually four) conserved cysteine residues. Based on the arrangement of these cysteine residues, two major chemokine groups have been categorised- the CC family with the first two cysteines adjacent to each other, and the CXC family that has one amino acid residue between the two cysteines. The chemokine (C-X-C Motif) ligand 14 gene (also known as BRAK, BMAC or Mip-2γ), located on human chromosome 5q31, is expressed as a 99 amino acid residue precursor protein, which is processed to a 77 amino acid mature protein with a molecular weight of 9.4 kDa having a highly basic isoelectric point of 9.9 (Fig. 1). CXCL14 was initially identified from breast and kidney cells in 1999 and is shown to be constitutively and widely expressed in normal tissue, especially in the epithelia [4, 5]. On the other hand, significant down-regulation or complete loss of expression is observed in many human cancer specimens and cancerous cell lines [5–8]. The primary amino acid sequence of CXCL14 is highly conserved in vertebrates [9]. Human and mouse CXCL14 differ in only two amino acid residues. As a non-ELR (Glu-Leu-Arg) CXC chemokine, CXCL14 has a short NH_2_-terminal end with only two amino acids (Ser-Lys) prior to the first typical Cysteine residue, while other CXC chemokines such as CXCL11 and CXCL12 have five or more residues in their NH_2_-terminal region (Fig. 1), which are essential for interaction with their cognate receptors [10, 11]. Another unique characteristic of CXCL14 worth mentioning is that it has a five consecutive amino acid insertion (^41^VSRYR^45^) not seen in other CXC chemokines, which was reported to be essential for its degradation in cancer cells [12]. These characteristics separate CXCL14 from other CXC chemokines, which could be critical determinants governing its functions.

## Cellular sources of CXCL14

**Fig. 2:**
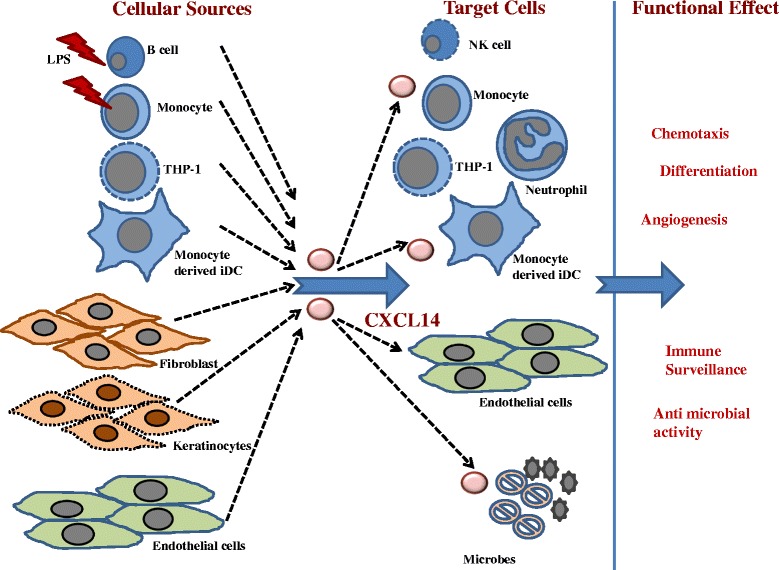
Schematic diagram showing the cellular sources of CXCL14, cells that have been demonstrated to express or release CXCL14 constitutively of following inflammatory cues; the target cells on which CXCL14 exerts its chemotactic activity, modulates differentiation or executes microbicidal actions thereby contributing to immune surveillance and immune defence

CXCL14 is expressed by a variety of immune and non-immune cells (Fig. 2) either constitutively or following distinct stimulatory influence. Freshly isolated resting human peripheral blood mononuclear cells do not exhibit CXCL14 expression at transcript (mRNA) level [6]. However following stimulation with lipopolysaccharide (LPS), CXCL14 mRNA is detected in monocytes and B cells, but not T cells [6]. The CXCL14 mRNA is also detected in THP-1 cell line and monocyte-derived dendritic cells (DCs), but not Jurkat cells [8]. Monocyte-derived immature dendritic cells (iDCs) secrete CXCL14 at low concentrations under basal conditions, which could be remarkably up-regulated by activin A [13]. Those leukocytes expressing CXCL14 all can be chemoattracted by CXCL14 indicating an immune surveillance role for CXCL14 [7, 8]. It is known that CXCL14 protein is highly expressed in healthy human epidermis and scattered cells of the dermis, while markedly less expression levels are observed in psoriatic and atopic dermatitis lesions [14]. Subsequently, it has been demonstrated that normal skin keratinocytes, dermal fibroblasts, dermal endothelial cells, and lamina propria cells but not epithelial cells in normal intestinal tissues are all sources of CXCL14, but not the immortal cell lines [15, 16]. The cutaneous CXCL14 is involved in the recruitment of CD14^+^ DC precursors and influences their differentiation to Langerhans cells [16]. Those CXCL14-producing fibroblasts are co-localized with macrophages, which suggests a role for CXCL14 in macrophage development [15]. Another study showed CXCL14, as a much highly expressed gene in taste buds of tongue from human, and it could be secreted into the saliva suggesting an immune surveillance function for this protein in line with the presence of leukocytes in human saliva [17, 18]. In addition, CXCL14 present on blood vessels in dermal plexus may be associated with its antiangiogenic effect in cancers [16, 19]. Therefore the chief cellular sources of CXCL14 as present in circulating blood cells, the skin barrier and saliva suggest its potential involvement in first line of innate and later acquired immune defence against pathogenic intrusions.

## Regulation of CXCL14 expression at transcript and protein levels

So far, the molecular mechanisms governing the expression of CXCL14 and CXCL14 mediated functions are not clear. It has been shown that CXCL14 expression could be inhibited by inflammatory stimuli such as TNF-α and LPS in epithelial tissues [4, 14]. Cigarette smoke condensate also could significantly suppress CXCL14 expression in normal human bronchial epithelial cells derived from healthy and non-smoking young male [20]. CXCL14 is significantly upregulated in inflamed joints of collagen-induced arthritis and its overexpression exacerbates arthritis in a mouse model, which is in line with the studies carried out in patients with rheumatoid arthritis [21, 22]. In another study, Padilla et al., revealed that CXCL14 mRNA is markedly upregulated in the left anterior descending coronary artery (LAD) and descending thoracic aorta of obese pig as a result of the high-fat diet feeding, which suggests a possible role of CXCL14 in the progress of atherosclerosis [23]. In cultured porcine endothelial cells, this upregulation is not LPS or NF-κB dependent [23]. Not only could the expression of CXCL14 transcript but CXCL14 also undergo post-transcriptional and post-translational regulations. For example as an anti-microbial peptide (AMP), CXCL14 is completely degraded by the cysteine proteinase SpeB from a strictly human pathogen *Streptococcus pyogenes* [24].

Since a lot of research over years has been done to study CXCL14 mediated effects in cancer, its expressional regulation has been explored in several types of carcinomas. RhoBTB2, an atypical member of the Rho family, as a tumour suppressor which is downregulated in a large proportion of breast and lung cancers [25], is able to positively regulate CXCL14 expression in normal and cancerous epithelial cells [26]. In breast cancer cells, reactive oxygen species (ROS) caused by interrupted mitochondrial respiratory chain increases CXCL14 expression through activation of the activator protein-1 (AP-1) [27]. As a consequence CXCL14 promotes cancer cell motility through elevated cytosolic Ca^2+^ released from the endoplasmic reticulum (ER) mediated through interaction between CXCL14 and inositol 1,4,5-trisphosphate receptor on the ER [27]. Ultraviolet irradiation or serum deprivation of oral squamous cell carcinoma cells increases CXCL14 expression by stimulating the phosphorylation of the mitogen-activated protein kinase (MAPK) p38, which reduces cell viability [28]. In this study, the suppression of ERK phosphorylation was also seen, in line with the previous report of MEK-ERK induced downregulation of CXCL14 by activation of epidermal growth factor receptor (EGFR) in oral carcinoma cells [29, 30]. It is also known that the phosphorylation of p38MAPK leads to activation of AP-1 [31], which was shown to be essential for elevation of CXCL14 expression as stimulated by ROS in breast cancer cells. These data suggest p38MAPK may play an important role through which different stress signal pathways regulate CXCL14 expression.

Due to its ill-defined receptor, intracellular signaling cascade initiated by CXCL14 still remains largely illusive. However it is known that CXCL14 can regulate calcium influx, NF-κB activity, activation of AP-1, and NOS1 as its intracellular molecular targets. Of interest, a post-translational mechanism for the loss of CXCL14 protein is reported in cancer and immortalized cell lines, but not in normal epithelial cells, which is regulated through ubiquitin-mediated, the 26 S proteasome participated degradation [12]. In human lung cancer cell line NCI-H460, which does not express potential target GPCRs for CXCL14, CXCL14 stimulates NF-κB activity to promote its proliferation and migration by binding to some glycoproteins on cell surface [32]. In gastric adenocarcinoma tissues, the unusually high methylation in CXCL14 promoter region contributes to the low expression levels of CXCL14 resulting in poor prognosis [33]. In prostate and breast cancer, it is reported that the tumor-supportive effect of fibroblast-derived CXCL14 depends on nitric oxide synthase NOS1 mediated signaling mechanisms [34, 35]. CXCL14 also facilitates invasiveness of pancreatic cancer cells by increasing nuclear NF-κB p65 levels [36]. In head and neck squamous cell carcinoma (HNSCC), CXCL14 expression and secretion and its anti-tumor effect is negatively regulated by RhoA/ROCK pathway. Therefore Fasudil, ROCK-specific inhibitor can stimulate CXCL14 expression [37]. In clear cell renal cell carcinoma, S100A6 (Calcyclin), as an oncogenic protein, negatively regulates CXCL14 expression, and knockdown of S100A6 promotes CXCL14-mediated apoptosis and promotes tumour progression [38]. In osteosarcoma, activation of Iroquois homeobox 1 (IRX1), as a prometastatic protein, directly increases CXCL14 expression, and promotes osteosarcoma metastatic activity via elevated CXCL14/NF-κB signaling [39]. These experimental evidences from cancer research could facilitate to build a research base for endeavours in infection and inflammation or immunological research to decipher the potential mechanisms by which expression and stability of CXCL14 could be modulated.

## Role of CXCL14 in mediating leukocyte migration and differentiation

As mentioned before, ambiguities still surround the identity of the cognate receptor for CXCL14. This makes it difficult to investigate the downstream intracellular signaling cascade of this chemokine completely. Recently, it has been demonstrated that CXCL14 specifically binds to CXCR4 with high affinity and supresses CXCL12-induced migration of THP-1 and human CD34^+^ bone marrow cells [40–42]. Another report indicates that CXCL14 in the conditioned medium from CD31^−^ side population (SP) cells promotes cell migration through CXCR4 on the migrated cells of the regenerated pulp tissue in an ectopic tooth transplantation model. Interestingly, CD31^−^ SP cells express CXCL12 only to a minimum extent [43, 44]. Thus conflicting experimental observations from different working groups have led to discrepancies regarding the chemotactic potential of CXCL14, which are summarized in Table 1. Unlike some other non-ELR CXC chemokines that are chemotactic for activated T cells [45, 46], CXCL14 fails to promote chemotaxis of naive or activated T cells [7, 8, 15]. Among other cells which have been indicated to display chemotaxis towards CXCL14 are CESS (human B-cell line), THP-1 (human monocyte leukemia cell line), human neutrophils, human and murine immature dendritic cells, human monocytes (especially prostaglandin E_2_ (PGE_2_) or forsklin-treated monocytes), activated human natural killer cells (NKs), and human uterine NKs [7, 8, 15, 45, 47]. Discrepancies might have risen due to difference in the methods of leukocyte isolation and use of CXCL14 protein from different sources by researchers, which include synthesised protein, murine CXCL14, CXCL14 present in conditioned media from transfected mammalian cell, and commercially available recombinant proteins from different sources [6–8, 16]. Since the availability of recombinant human CXCL14 (rCXCL14), it has been demonstrated that CXCL14 effectively promotes chemotaxis of only immature, but not mature DCs both *in vivo* and *in vitro* [19, 48]. In tumour tissues, the loss of CXCL14 is associated with reduced infiltration of DCs, which could otherwise elicit a specific antitumor immunity [48]. CXCL14 can further promote DC activation accompanied by up-regulation of NF-κB activity [48]. Moreover, Schaerli et al.,. demonstrated a role of CXCL14 and the epidermal environment in selectively recruiting blood CD14^+^ DC precursors to be differentiated into epidermal Langerhans cells under steady-state condition by using a human epidermal tissue model, which is consistent with the constitutive expression of CXCL14 in the epidermis of healthy skin [15, 16]. However, a series of synthetic CXCL14 variants with amino-terminal extensions show no chemoattractant effect on CD14^high^ monocytes [16]. Presumably, the amino terminus of CXCL14 might be important for its interaction with the chemotactic receptor on target cells which respond to CXCL14. However, no significant difference is observed in the numbers of monocytes, macrophages and DCs in blood, bone marrow, and secondary lymphoid organs among CXCL14 knockout mice and their wild type counterparts, or among the CXCL14-transgenic (Tg) mice [22, 49]. Interestingly, enhanced T cell activation and proliferation was shown in CXCL14-Tg mice compared to the control mice, despite no alteration in the number of lymphocytes [22]. Very recently, significantly reduced number of uterine NK cells was demonstrated in CXCL14^−/−^ pregnant uterus as compared to CXCL14^+/−^ pregnant uterus, which is in line with the chemoattractive effect of CXCL14 on human uterine NK cells as observed *in vitro* [45, 50]. These observations indicate that CXCL14 is not indispensable for maintaining chemotaxis or steady state kinetics and turnover of immune cells *in vivo* and might have other chemokines complementing its functions. Among all 48 human chemokines, only CXCL12 (also known as SDF-1α) matches the high degree of CXCL14 conservation in vertebrate species [51]. Both are known as evolutionary ancient chemokines due to their highly conservative sequence throughout different vertebrate classes and their homeostatic roles, which suggests that CXCL14, evolved with CXCL12, might have the potential to regulate the function of the CXCL12-CXCR4 signaling axis or play a compensatory role when CXCL12 is absent [9, 40, 43, 52, 53]. However, other report demonstrates that CXCR4 alone is not sufficient to initiate CXCL14-mediated chemotactic response. CXCL14 also fails to influence the CXCL12-CXCR4 mediated signaling axis in Jurkat cells and HEK293 cells transfected with CXCR4 [54]. So other potential GPCR or signaling mediators still need to be identified for CXCL14-specific biological activities in co-operation with CXCR4.

**Table 1:** Cellular sources, target cells and potential functional significance of CXCL14 to immune functions

Cells or tissues expressing CXCL14 as mRNA or protein	Target cells for CXCL14	Functional effects of CXCL14	Sources of recombinant CXCL14 protein	Reference
mRNA detected in human heart, brain, placenta, lung, liver, skeletal muscle, kidney, and pancreas; colon adenocarcinoma cell SW 485, and melanoma cell MDA-MB-435		No *in vitro* effect on human endothelial cell proliferation or tubule formation; No chemotactic effects on human or murine T cells, B cells, monocytes, NK cells, or granulocytes	Synthesized human CXCL14	1999 Hromas et al. [5]
mRNA detected in mouse brain, ovary, lung, and muscle; Human intestine, colon, kidney, liver, spleen, thymus, placenta, brain, pancreas, skeletal muscle, heart, cervix, uterus, and breast	B cells, macrophages, CESS (human B cell line), A20 (murine B cell line), THP-1 (human monocyte leukemia cell)	Chemoattraction of CESS and THP-1 cells; Inflammation induction *in vivo* in Nude mice	Synthesized murine CXCL14	2000 Sleeman et al. [7]
mRNA detected in human kidney, intestine, brain, placenta, skeletal muscle, liver, spleen, thymus, and pancreas; very faint expression in testis, ovary, heart, and lung.	Human neutrophils and monocyte-derived DC	A strong chemoattractant for human neutrophils, and weaker for human DC	Human CXCL14 in supernatant from transfected 293 cells	2000 Cao et al. [8]
Epithelium of tubules of mouse kidney; hepatocytes in mouse liver; monocyte-derived dendritic cell (DC); THP-1				
mRNA in human skin, kidney, intestine, spleen, colon, muscle, liver, brain, placenta, thymus, breast, exocervix, ovary and heart; squamous epithelium; oral epithelial cells, epidermal keratinocytes; LPS activated B cells and monocytes; inflammatory and stromal cells adjacent to carcinomas		Potential role in host-tumor interactions		2000 Frederick et al. [6]
mRNA in human epithelial layer of intestine, kidney, stomach, colon, appendix, trachea; Skin keratinocytes, dermal fibroblasts, lamina propria cells in intestine; HaCaT (human keratinocyte cell line)	Freshly isolated monocytes (weak), monocytes treated with prostaglandin E_2_ (PGE_2_) or forskolin (strong)	Monocyte chemoattractant; potential role in macrophage development; CXCL14 signals through Bordetella pertussis toxin-sensitive receptor in PGE_2_-treated monocytes	Synthesized human CXCL14	2001 Kurth et al. [15]
CXCL14 protein expression in human: Suprabasal layers of tongue mucosa, stromal cells adjacent to tumors	human endothelial cells, monocyte-derived immature dendritic cells (iDCs)	Potent inhibitor of chemotaxis for human endothelial cells and *in vivo* angiogenesis	Recombinant human CXCL14	2004 Shellenberger et al. [19]
CXCL14 protein in human: Oral squamous epithelium	Human monocyte-derived iDCs	Stimulation of iDCs migration and maturation, and NF-κB activation	Recombinant human CXCL14	2005 Shurin et al. [48]
Protein expression in human: blood vessels in dermal plexus and epidermal keratinocytes of skin	CD14^+^ DC precursors derived from CD34^+^ hematopoietic progenitor cells (HPCs) and blood CD14^+^monocytes	Stimulation of CD14^+^ monocyte migration, possible contribution to the differentiation of CD14^+^ DC precursors into Langerhans cell-like cells in epidermal tissue under steady-state condition	Recombinant human CXCL14 and CXCL14 purified from supernatant of primary keratinocyte culture	2005 Schaerli et al. [16]
	Activated human natural killer(NK) cells; an IL-2-dependent natural killer leukemia cell line; monocyte-derived iDCs	Stimulation of activated human NK cells and iDCs; no effect on proliferation or cytotoxic activity of normal human NK cells	CXCL14 synthetic peptide, CXCL14 expressed in bacterial vector and HPLC-purified; recombinant eukaryotic CXCL14	2006 Starnes et al. [47]
DCs stimulated with activin A	Human and murine iDCs	Mediator for activin A-induced migration of iDCs	Recombinant human CXCL14	2009 Salogni et al. [13]
Protein in human: glandular epithelial cells in endometrium in the secretory phase of menstrual cycle	Human uterine natural killer (uNK) cells	Stimulation of uNK cell migration during the secretory phase of the cycle	Recombinant human CXCL14	2010 Mokhtar et al. [45]
	Human THP-1 cells and iDCs	The chemotactic effect on THP-1 and iDCs	Recombinant human CXCL14	2010 Tanegashima et al. [64]
	PGE_2_-treated THP-1, *Pseudomonas aeruginosa, Streptococcus mitis*, and *Streptococcus pneumoniae*	Induction of THP-1 migration; antimicrobial activity to facilitate bacterial clearance in mouse lung	Recombinant human CXCL14	2015 Dai et al. [62]

## Antimicrobial activity of CXCL14

Although human skin is constantly exposed to a variety of microorganisms, normally no signs of infection or excessive microbial growth are shown on our healthy body surfaces [55]. Apart from the physical barrier, the existence of abundant antimicrobial peptides (AMPs) contributes to prevent a wide spectrum of bacteria, yeast, and some enveloped viruses from infiltrating into the live part of the epidermis [56–59]. Defensins and cathelicidin LL-37 are the main AMPs expressed in human skin [58, 59]. Interestingly, CXCL14 shares striking common structural characteristics with them, including large, positively charged patches on its molecular surface, three anti-parallel β-strands shown in β-defensin, and a C-terminal α-helix that is typical for cathelicidin LL-37 (Fig. 1) [60]. CXCL14 and human β-defensin both show bactericidal activity against Gram-positive coccoid *Finegoldia magna* residing in skin epidermis and the virulent pathogen *Streptococcus pyogenes* at similar concentrations [4]. Thus, in addition to its chemoattractant effects on immune and inflammatory cells, recombinant CXCL14 demonstrates direct antimicrobial effects against *Escherichia coli* and *Staphylococcus aureus*, but not *Candida albicans* at 10μg/ml [61]. Characteristic topological formation of a large positive electrostatic patch on the molecular surface of CXCL14 is required for its antimicrobial activity as deciphered in comparison of antimicrobial chemokines with non-antimicrobial chemokines. Subsequently, CXCL14 has demonstrated to have a broad range of antimicrobial effects against cutaneous Gram-positive bacteria and *Candida albicans* as well as the Gram-negative enterobacterium *Escherichia coli*, which could not be counteracted by increased salt concentrations and skin-typical pH conditions [14]. Those antimicrobial activities exhibited by both human and murine CXCL14 could be neutralized by anti-CXCL14 mAb [14]. Since CXCL14 is constantly produced in the epidermis and dermis of healthy human skin and other epithelial tissues but is markedly decreased in the presence of inflammation and disease, it has been proposed that CXCL14 plays a unique role in antimicrobial immunity prior to the establishment of inflammation [4, 14, 15].

Moreover, CXCL14 also plays prominent anti-microbial role in animal models of respiratory tract infection. It executes bactericidal actions against *Pseudomonas aeruginosa*, *Streptococcus mitis*, and *Streptococcus pneumoniae* partially via bacterial membrane depolarization, damage and via its binding affinity for bacterial DNA [62]. In this study, the authors showed that the disulphide bonds and the C-terminal α-helix of CXCL14 do not seem to be responsible for its antimicrobial activity [62], although a high proportion of cationic amino acids in the α-helix part might be involved in the disruption of bacterial membranes by binding with negatively charged molecules in bacterial membranes. The N-terminal region of CXCL14 is critical for its antimicrobial activity, at least, against Gram-negative bacteria such as *E. coli* and *Pseudomonas aeruginosa*, while the CXCL14 fragment showed no chemoattractant effect for human blood monocytes at micromolar concentrations [62]. To substantiate the antibacterial effects of CXCL14 both CXCL14 mRNA and protein expression levels are shown to be increased following *Pseudomonas aeruginosa* infection in the lung tissue of wild type mice. On the other hand, CXCL14-deficient mice exhibit reduced bacterial clearance in the lungs infected with *Streptococcus pneumoniae* but not *Pseudomonas aeruginosa* [62]. This is the first *in vivo* demonstration that CXCL14 has a role in host defence against lung infections induced by Gram-positive bacteria. The exact antimicrobial mechanism for CXCL14 remains to be deciphered. Nevertheless, it might be considered that classical AMPs like defensins and cathelicidin exert less influence on Gram-positive bacteria compared with Gram-negative bacteria. For instance, cathelicidin deficient mice showed an impaired lung bacterial clearance in response to Gram-negative *Pseudomonas aeruginosa* [63]. These studies suggest the possibility that different structural properties of CXCL14 and antimicrobial mode of action might be associated with extermination of Gram-negative and Gram-positive bacterial species. The molecular regions of CXCL14 acting as an AMP and a chemoattractant appears to be different, and CXCL14 shows a non-redundant role as an AMP in lung infections. Future studies utilizing animal models of systemic infection like sepsis could reveal the antimicrobial potential of CXCL14 not only as a therapeutic alternative administered to these models but decipher the role of active immune cell derived CXCL14 in providing antimicrobial defence under physiological conditions.

## Conclusions

During recent years of endeavours in the ever expanding field of chemokine research CXCL14 has emerged as a potential mediator of inflammatory processes and immune response as highlighted in this review. It also bears the potential to influence other chemokine functions particularly that of CXCL12. Identification of its cognate receptor in the coming years would help reveal the molecular mechanisms by which CXCL14 might influence immune response also uncover more cellular targets under diverse pathophysiological conditions which involve immune cells.
